# The effect of task symmetry on bimanual reach-to-grasp movements after cervical spinal cord injury

**DOI:** 10.1007/s00221-018-5354-8

**Published:** 2018-08-21

**Authors:** Laura Britten, R. O. Coats, R. M. Ichiyama, W. Raza, F. Jamil, S. L. Astill

**Affiliations:** 1 School of Biomedical Sciences, Faculty of Biological Sciences, University of Leeds, Leeds, LS2 9JT UK; 2 Faculty of Medicine and Health, School of Psychology, University of Leeds, Leeds, LS2 9JT UK; 3 Yorkshire Regional Spinal Injuries Centre, Pinderfields General Hospital, Aberford Road, Wakefield, WF1 4DG UK

**Keywords:** Spinal cord injury, Bimanual control, Reaching, Grasping

## Abstract

Injury to the cervical spinal cord results in deficits in bimanual control, reducing functional independence and quality of life. Despite this, little research has investigated the control strategies which underpin bimanual arm/hand movements following cervical spinal cord injury (cSCI). Using kinematics and surface electromyography this study explored how task symmetry affects bimanual control, in patients with an acute cSCI (< 6 m post injury), as they performed naturalistic bimanual reach-to-grasp actions (to objects at 50% and 70% of their maximal reach distance), and how this differs compared to uninjured age-matched controls. Twelve adults with a cSCI (mean age 69.25 years), with lesions at C3–C8, categorized by the American Spinal Injury Impairment Scale (AIS) at C or D and 12 uninjured age-matched controls (AMC) (mean age 69.29 years) were recruited. Participants with a cSCI produced reach-to-grasp actions which took longer, were slower, less smooth and had longer deceleration phases than AMC (*p* < 0.05). Participants with a cSCI were less synchronous than AMC at peak velocity and just prior to object pick up (*p* < 0.05), but both groups ended the movement in a synchronous fashion. Peak muscle activity occurred just prior to object pick up for both groups. While there seems to be a greater reliance on the deceleration phase of the movement, we observed minimal disruption of the more impaired limb on the less impaired limb and no additional effects of task symmetry on bimanual control. Further research is needed to determine how to take advantage of this retained bimanual control in therapy.

**Electronic supplementary material:**

The online version of this article (10.1007/s00221-018-5354-8) contains supplementary material, which is available to authorized users.

## Introduction

Many activities of daily living require the use of both hands simultaneously, and given that injury to the cervical spinal cord (cSCI) often results in bilateral deficit (Spooren et al. 2009), bimanual movements are often impaired. Animal models have indicated that bimanual movements arise due to anatomical and neuronal crosstalk, particularly of the corticospinal tract (CST) (Rosenzweig et al. 2009) and interhemispheric connections within the brain (Cardoso de Oliveira et al. 2001). Therefore, injury to the cervical spinal cord, and specifically the CST, will result in deficits in bimanual control. However, spared fibres of the CST, may be a mechanism for recovery as neuroplasticity in the CST have previously been shown to induce improvements in function (Rosenzweig et al. 2009, 2010; Krajacic et al. 2010). Spinal interneurons (Takei and Seki 2010) and propriospinal neurons (Sasaki et al. 2004) have also been shown to innervate upper limb muscles in bimanual movement and those spared fibres unaffected by the primary cSCI may also act as a mechanism for recovery (Lemon 2008).

Neuroplasticity of spared fibres can be maximised during therapy to yield improvements in function (Marsh et al. 2011). Bimanual therapy after cSCI has been shown to improve bimanual upper limb function and these improvements have been in individuals with a chronic cSCI (Hoffman and Field-Fote 2010, 2013), suggesting that further gains in function could occur if bimanual therapy is given in the acute stages of injury when the greatest neuroplasticity occurs (Curt et al. 2008). However, there has been little research that has quantified how control of bimanual movements changes after cSCI (Cacho et al. 2011; Calabro and Perez 2016).

In the non-injured population, a large amount of research have focused on how symmetrical and asymmetrical bimanual tasks influence kinematic characteristics and interlimb synchrony. Although some research has found evidence for temporal synchrony in asymmetric aiming and prehension tasks (Kelso et al. 1979; Jackson et al. 1999), more recently the general consensus is for temporal asynchrony between the limbs (Riek et al. 2003; Bingham et al. 2008; Mason and Bruyn 2009; Miller and Smyth 2012), which is thought to occur due to visual constraints, i.e. the inability to fixate both objects and limbs at one time (Riek et al. 2003; Mason and Bruyn 2009).

Calabro and Perez (2016) found that during asymmetric bimanual reaches to objects of different sizes, interlimb synchrony was reduced in individuals with a cSCI as the more impaired limb had detrimental effects on the less impaired limb, specifically when opening and closing the hand (Calabro and Perez 2016). However, this study solely focused on the influence of object size on prehension. Other characteristics also play a role in affecting reach-to-grasp movements. Research shows that manipulation of object distance also affects the kinematics of the transport and grasp phase of a prehensile action (Fitts 1954; Jakobson and Goodale 1991; Bootsma et al. 1994). Furthermore, given that neurologically it is hypothesised that interference at the execution level, and thus degree of synchrony observed, may be more pronounced for arm transport than grasp formation (Donchin et al. 1999; Heuer et al. 2001) it is important to document the impact of object distance on the control of bimanual reach to grasp movements in individuals with cSCI, as this could have a direct impact on rehabilitation paradigms. Recently, we have shown that when individuals with an acute cSCI made reaching movements to symmetrical objects (i.e. placed at the same distance) there seems to be little detrimental effect of the more impaired limb on the less impaired limb (Britten et al. 2017). However, the performance of many everyday activities requires the completion of asymmetric but coordinated movements with the two hands to achieve the task goal, e.g. reaching for two objects placed at different distances. Investigating how individuals with cSCI control asymmetric reach to grasp movements, whereby object distance is manipulated, has yet to be undertaken, resulting in an important gap in our knowledge of movement control in this clinical population.

Age deficits in bimanual coordination have led to suggestions that older adults may rely more heavily on visual feedback during bimanual movements than younger adults (Coats and Wann 2012; Britten et al. 2017), which could be due to deficits in processing sensory information (Sosnoff and Newell 2006). This reliance on visual feedback and thus reduced synchrony between the limbs could be increased in individuals with a cSCI due to their already existing motor and sensory dysfunction (Lemon 2008). Furthermore, it has been reported that the mean age of an individual having a cervical level SCI has recently increased (Thompson et al. 2014), with the suggestion that this increase is due an injury sustained after a fall (Devivo 2012), which are growing increasingly common for older adults. Thus, the reliance on visual feedback due to the aberrant sensory information after a cSCI could also be exacerbated by the ‘natural’ ageing process in these individuals. In addition, when the reach to grasp task is asymmetric in nature, the need to visually attend to one object then another when at different distances (Riek et al. 2003), could also drive decreased synchronicity between the limbs.

Previous research has shown that individuals with a cSCI have an impaired ability to utilise cross facilitation, whereby contraction of upper limb muscles on one side of the body increases excitability of muscles on the other side of the body (Bunday and Perez 2012). Therefore, participants with a cSCI may show differences in timing of peak muscle activity patterns between the more and less impaired limb, which may be exacerbated in asymmetrical trials when each limb is moving a different distance. We have previously suggested (Britten et al. 2017) that prolonged movement duration during the transport phase of the movement could be related to weakness of the proximal arm muscles, particularly the triceps brachii (Gronley et al. 2000), and the development of novel muscle activity patterns, e.g. activation of the shoulder complex to produce passive elbow extension, to compensate for weakened or lost muscle function (Koshland et al. 2005). This contention was also supported recently by Lei and Perez (2017) who noted that maximal voluntary muscle contractions in proximal arm muscles were significantly lower than non-injured controls (Lei and Perez 2017). It is clearly important therefore, that further investigation of reach to grasp symmetries in individuals with a cSCI utilises surface EMG as this information might be relevant to the design of training paradigms to enhance muscle strength and reach to grasp function.

The aim of this study was to compare symmetric and asymmetric bimanual movements (by manipulating object distance) in individuals with cSCI, and how this differs to non-injured controls, age matched due to the effects of ageing on reach-to-grasp movements. Based on previous work, we expected that (1) participants with a cSCI would produce movements of a longer duration, with a lower peak velocity and less smooth movements than age-matched controls (AMC), (2) participants with a cSCI would produce movements with greater interlimb asynchrony than AMC and this difference may be exacerbated during asymmetrical conditions, and (3) participants with a cSCI would show different peak muscle activity patterns to AMC.

## Methods

### Participants

Twelve inpatients who were in the acute stage (< 6 months since injury) of recovery were recruited from two UK Spinal Injuries Centres (Table 1 shows participant characteristics). The inclusion criteria were as follows; > 18 years old, had a SCI at the cervical level, could understand and follow verbal instructions, could give written consent and had no history of additional neurological impairment. Participants that could not meet these criteria were excluded from the study. The preferred limb of participants with a cSCI was determined as the less impaired limb when completing the Chedoke Arm and Hand Inventory-9 (Barreca et al. 2006). Twelve age-matched control participants (AMC) were recruited from the local community (mean age 69.29 ± 7.32 years; 10 right handed; 7 Female). Ethical approval was sought from the University of Leeds and local NHS Ethical Review Committees. Ethical procedures conformed to the declaration of Helsinki, and all participants gave written informed consent.

**Table 1:** Participants’ characteristics of individuals with cSCI

cSCI subject	Age (years)	Gender (M/F)	Level	AIS score	Aetiology	Time since injury (weeks)	More affected limb	CAHAI-9	Maximal reach distance (cm)
1	68	M	C5	D	T	7	L	60	39.5
2	67	M	C7	C	T	17	R	56	38
3	57	M	C8	C	NT	11	L	–	32
4	79	F	C6	D	T	23	L	62	28
5	69	M	C5/6	–	T	8	L	58	28.2
6	79	M	C5	C	NT	9	L	63	37.2
7	73	M	C3/4	C	T	18	R	52	31
8	65	M	C3–6	D	T	15	R	44	26
9	65	M	C5	C	T	14	L	56	42
10	56	M	C5	C	NT	21	L	–	45
11	67	M	C4	D	T	6	L	63	40
12	86	M	C3/4	D	NT	10	L	63	59

### Experimental set-up

The experimental set-up was similar to previous research (Britten et al. 2017). Participants sat in a chair or their wheelchair (with their hips and knees at 90°) at a height adjustable table. Before testing, maximal reach distance for each participant was calculated as the maximum distance between the edge of the height adjustable table and where each participant could reach with their fingertips of both arms (arms fully extended) on the table, with their back against the chair/wheelchair (to minimise trunk involvement). The chair remained in the same place relative to the height adjustable table throughout the testing session. Maximal reach distance was recorded to standardize the object placement relative to participant ability.

We investigated kinematics and interlimb synchrony when reaching bimanually in symmetric and asymmetric conditions, whilst object size remained constant (30 mm × 30 mm × 18 mm). Objects (plastic blocks) were placed at 50% (near) or 70% (far) of each participant’s maximal reach distance and kept 20 cm apart (10 cm at either side of the participant’s midline). The bimanual reach-to-grasp movements were performed using a precision grip at a self-selected, comfortable speed after a verbal ‘go’ command. Once task familiarisation had taken place participants performed 24 trials in four blocks (two symmetrical and two asymmetrical); eight trials with the preferred/less impaired limb (P/LI) and non-preferred/more impaired limb (NP/MI) both reaching to the near objects (condition one; NN), eight trials with the P/LI limb and NP/MI limb reaching to the far objects (condition two; FF), eight trials with the P/LI limb reaching to the near object and NP/MI limb reaching to the far object (condition three; NF), eight trials with the P/LI limb reaching to the far object and NP/MI limb reaching to the near object (condition four; FN). The order of trials was blocked and pseudo-randomised between participants. All participants completed the required number of trials, without the need for additional trunk support or compensatory trunk movements and had full view of the arms/hands and the objects during each trial.

**Fig. 1:**
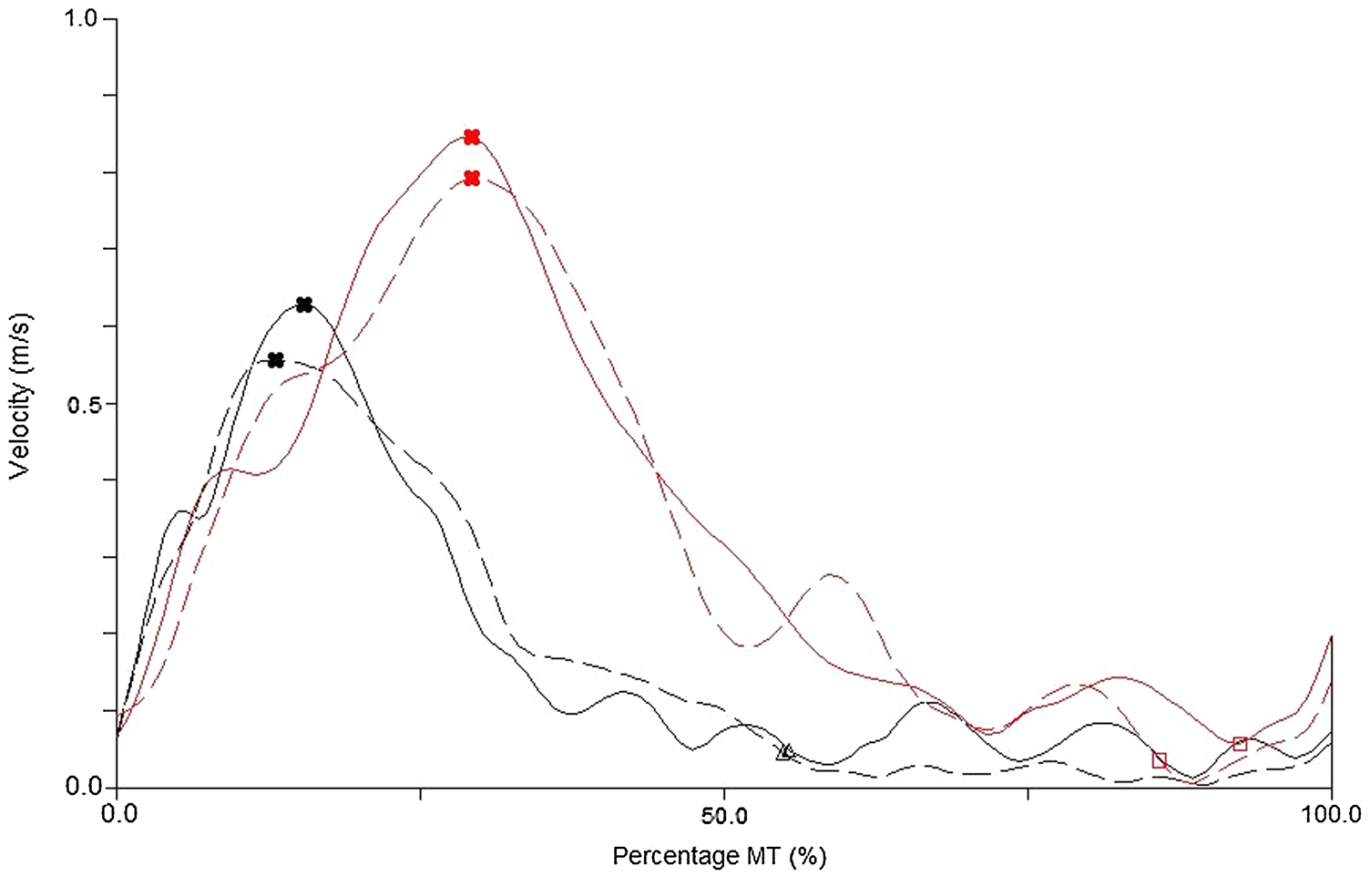
Examples of kinematic velocity profiles for a participant with a cSCI [dashed (LI limb) and solid (MI limb) black lines] and an AMC [dashed (P limb) and solid (NP limb) red lines], in condition one (Near Near) [graphed between movement onset (0%) and the end of the movement (100%)]. The cross markers show the ToPV, triangle markers show FAP_start_ for cSCI and square markers show FAP_start_ for AMC

Reflective markers were placed on the right and left medial styloid process, the distal portion of the index fingers and thumbs of both hands, and the objects and recorded with a 5-camera motion analysis system (Proreflex, Qualisys, Sweden) sampling at 120 Hz. Data were filtered using a low-pass Butterworth filter with a cut-off frequency of 10 Hz (Bootsma et al. 1994; Paulignan et al. 1997), and were then analysed using Visual3D software (C-motion, USA). Kinematic landmarks were identified on the tangential velocity profiles using a custom-written program (in Visual3D) and confirmed by visual analyses of the velocity profiles (see Fig. 1 for examples of velocity profiles).

Muscle activity was measured using the Noraxon Telemyo 2400T with G2 miniature wireless receiver EMG system testing at 1500 Hz, which was synchronized with the Proreflex Motion Capture System using an external trigger. Standard surface electrodes were used and the skin was prepped using alcohol based swabs and Nu-Prep skin preparation gel to prevent any resistance. Electrodes were placed on the anterior deltoid, biceps brachii, lateral head of the triceps brachii and the extensor digitorum superficialis. These muscles were chosen due to their contribution to the task, they are representative of cSCI to C5–C8, and have shown differing levels of paresis depending on the skeletal level of the injury (Janssen-Potten et al. 2008; Mateo et al. 2015). The electrodes were placed parallel to the direction of the muscle fibres and activity of the muscles was checked prior to data collection to ensure correct electrode placement, i.e. the triceps were checked using extension of the elbow and biceps using flexion of the elbow. For six of the participants (three participants with a cSCI and three older adults) there is missing EMG data due to technical difficulties. Visual 3D was used to first filter the data using a high-pass filter with a cut-off frequency of 20 Hz, second, the data were then full wave rectified, and third, a low-pass filter with a cut off frequency of 5 Hz was applied (Bonnefoy et al. 2009). The timing of peak muscle activity as a percentage of movement time was then identified for each muscle and each limb for each participant.

### Data analysis

We calculated several parameters related to the transport and grasp phases in line with past research (Coats and Wann 2011) (Britten et al. 2017). Movement onset (MO) was defined as when the velocity of the wrist reached 50 mm/s, and movement end (END) once the object moved 50 mm in the vertical direction (*z*). From MO and END, the following were then calculated; (1) movement time (MT): the duration of time between MO and END, (2) peak velocity (PV): the maximal velocity of the wrist during MT, (3) time of peak velocity (ToPV): the timing of PV as a percentage of MT, (4) percentage of MT spent decelerating (DT): the time between ToPV and END expressed as a percentage of total MT, (5) final adjustment phase (FAP): the time between the velocity of the wrist reaching 50 mm/s during the deceleration phase (FAP_start_) and END, as a percentage of total MT (see Fig. 1 for examples of velocity profiles). (6) Movement smoothness was examined using the number of zero crossings (Steenbergen and Van Der Kamp 2004) in the acceleration profile of the approach phase (NOAA: ToPV to FAP_start_) and final adjustment phase (NOAF: FAP_start_ to END). ‘Interlimb synchrony’ (7) was calculated as the absolute difference in time between the P/LI and NP/MI limbs at MO, ToPV, FAP_start_ and END.

From the markers on the thumb/s and index finger/s of each hand, the following were calculated: (1) maximum grasp aperture (MGA), the largest distance between the index finger and thumb during MT; (2) the time at which this MGA occurred during MT expressed as a percentage of total MT (tMGA); (3) the coupling of the grasp and transport phase (TrG) calculated as the time of peak deceleration minus the time of MGA, with a smaller value indicating greater coupling.

With regards to surface EMG, the time difference (subtracting one from the other) between timing of peak muscle activity (as a percentage of movement time) and timing of kinematic events (as a percentage of movement time) (MO, ToPV, FAP_start_ and the END) was calculated. This was to establish muscle patterns throughout the kinematic reach-to-grasp movement, which has not been previously investigated within the cSCI population. A positive value indicates that peak muscle activity occurred after the kinematic event and a negative value indicates that peak muscle activity occurred before the kinematic event. Finally, the time difference between the time of peak triceps brachii activity (agonist) and lowest biceps brachii activity (antagonist) was quantified to assess the agonist–antagonist muscle activity during the reach-to-grasp movement.

### Statistical analysis

Data were examined using group (cSCI, AMC) × condition (NN, FF, NF, FN) x limb (P/LI, NP/MI) repeated-measures ANOVAs for each variable. Statistical significance was set at *p* < 0.05 and significant main effects were investigated using pairwise comparisons with Bonferonni adjustments. All significant interactions were explored using the appropriate inferential statistics. When sphericity could not be assumed *F* and *p* values were generated using the Greenhouse–Geisser correction. Interlimb synchrony was examined using group × condition repeated-measures ANOVAs as limb was no longer a variable.

## Results

**Fig. 2:**
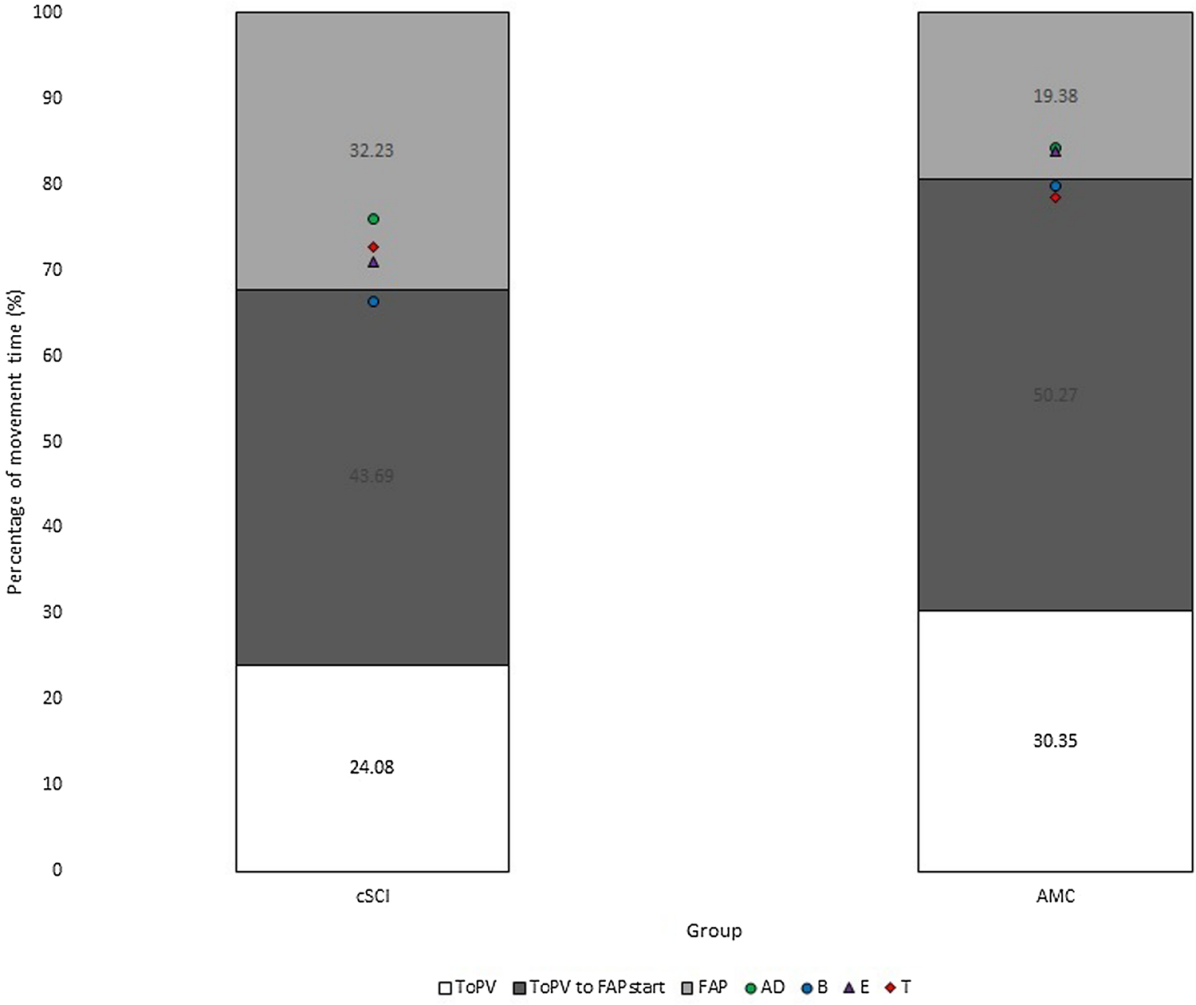
Normalised time [percentage of MT (%)] spent in each phase of the movement [white = MO (0%) to ToPV, dark grey = ToPV to FAP_start_ and light grey = FAP (FAP_start_ to END (100%)], and timing of peak muscle activity [percentage of MT (%)] for participants with a cSCI and AMC [green circle = AD/anterior deltoid, blue circle = B/biceps brachii, purple triangle = E/extensor digitorum superficialis, red diamond = T/triceps brachii]

**Fig. 3:**
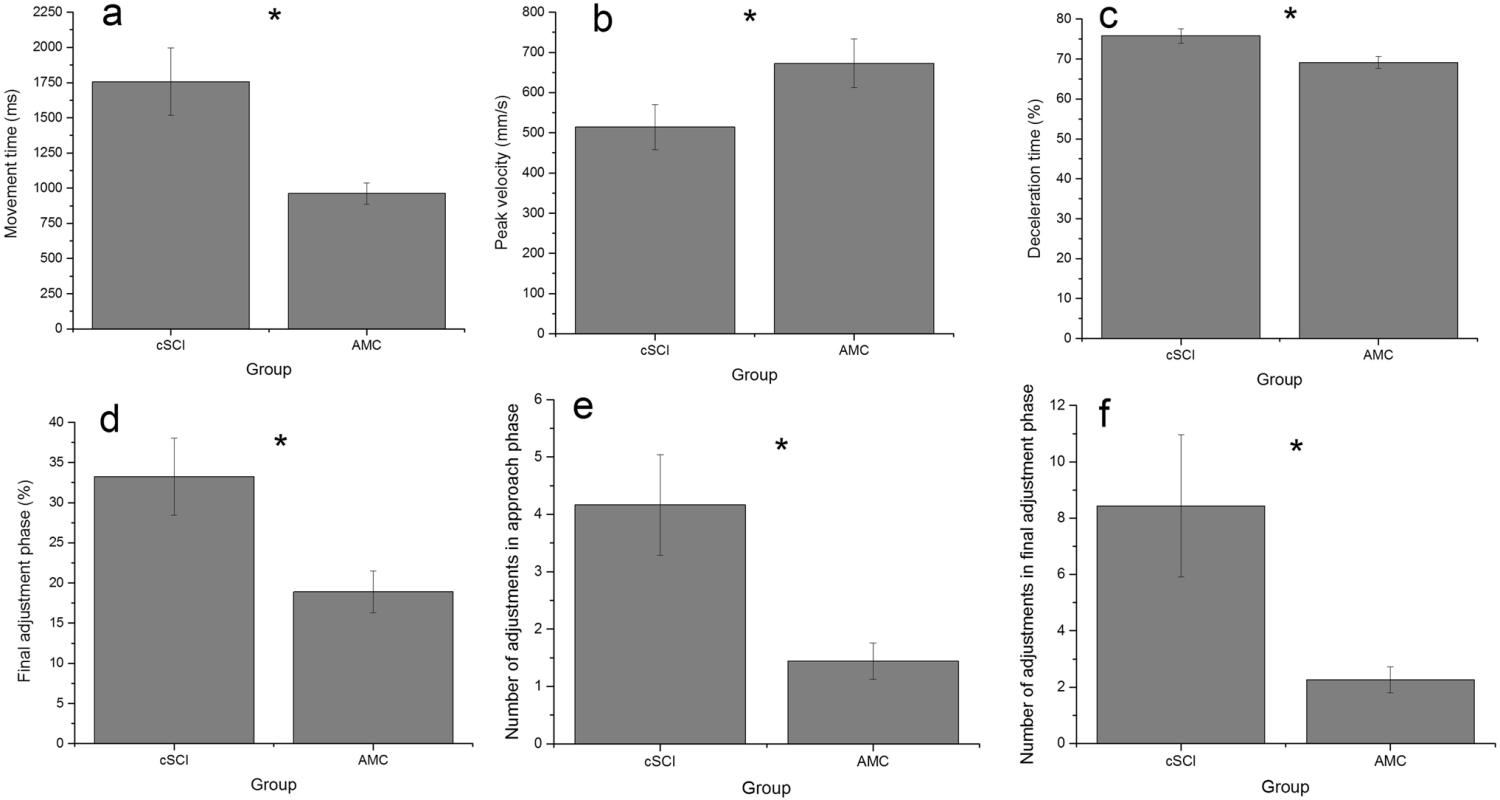
Group means (± standard error) for movement time (**a**), peak velocity (**b**), proportion of movement time spent decelerating (**c**), proportion of movement time spent in the final adjustment phase (**d**) and number of adjustments in the approach (**e**) and final adjustment phase (**f**) (*denotes a significant group difference)

All means (± standard error) are available for each kinematic variable in Supplementary Table 1, but for reasons of brevity and focussing on our main question of interest (group differences) only group means are presented in Figs. 2 and 3.

### Transport phase; MT, PV, DT, FAP, NOAA, NOAF

Participants with a cSCI (*m* = 1757 ms) produced movements of a longer duration than AMC (*m* = 961 ms) [*F*(1,29) = 9.06, *p* < 0.01, *η*^2^ = 0.48] and reached a lower PV [*F*(1,19) = 5.83, *p* < 0.05, *η*^2^ = 0.31] (cSCI 513 mm/s, AMC 672 mm/s) (Fig. 3a, b). Participants with a cSCI (DT 75.80%, FAP 33.22%) also spent a longer proportion of the movement decelerating [*F*(1,19) = 7.73, *p* < 0.05, *η*^2^ = 0.29] and in the final adjustment phase [*F*(1,19) = 5.97, *p* < 0.05, *η*^2^ = 0.31] (Figs. 1, 2, 3c, d) compared to AMC (DT 69.12%, FAP 18.89%) and made more adjustments than AMC (NOAA 1.44 vs 4.16, NOAF 2.27 vs 8.44) in both the approach [*F*(1,19) = 10.34, *p* < 0.01, *η*^2^ = 0.54] and final adjustment phase [*F*(1,19) = 4.34, *p* = 0.05, *η*^2^ = 0.19] (Fig. 3e, f).

For PV [*F*(3,17) = 89.42, *p* < 0.001, *η*^2^ = 0.94], FAP [*F*(3,17) = 12.28, *p* < 0.001, *η*^2^ = 0.68] and NOAF [*F*(3,17) = 4.26, *p* < 0.05, *η*^2^ = 0.43], a significant condition by limb interaction emerged. These interactions emerged as in asymmetrical conditions (condition three NF and four FN) the limb reaching to the far object (NP/MI limb in condition three = 686.58 mm/s, P/LI limb in condition four = 670.62 mm/s), reached a higher peak velocity than the limb reaching to the near object (P/LI limb in condition three = 599.54 mm/s, NP/MI limb in condition four = 539.05 mm/s). Additionally, in asymmetrical conditions, the limb reaching to the near object spent a longer proportion of the movement in the final adjustment phase [condition three (NF) P/LI = 31.02% NP/MI = 22.08%, condition four (FN) P/LI = 24.58% NP/MI = 30.26%] and made more adjustments [condition three (NF) P/LI = 5.33 NP/MI = 3.96, condition four (FN) P/LI = 4.47 NP/MI = 6.01] in this phase compared to the limb moving to the far object.

### Grasp phase: MGA, tMGA, TrG

There was no significant main effect of group, condition or limb for MGA or tMGA. However, participants with a cSCI (*m* = 334 ms) produced less coupled transport and grasp phases compared to AMC (*m* = 114 ms) [*F*(1,19) = 8.49, *p* < 0.01, *η*^2^ = 0.45].

### Interlimb synchrony

**Fig. 4:**
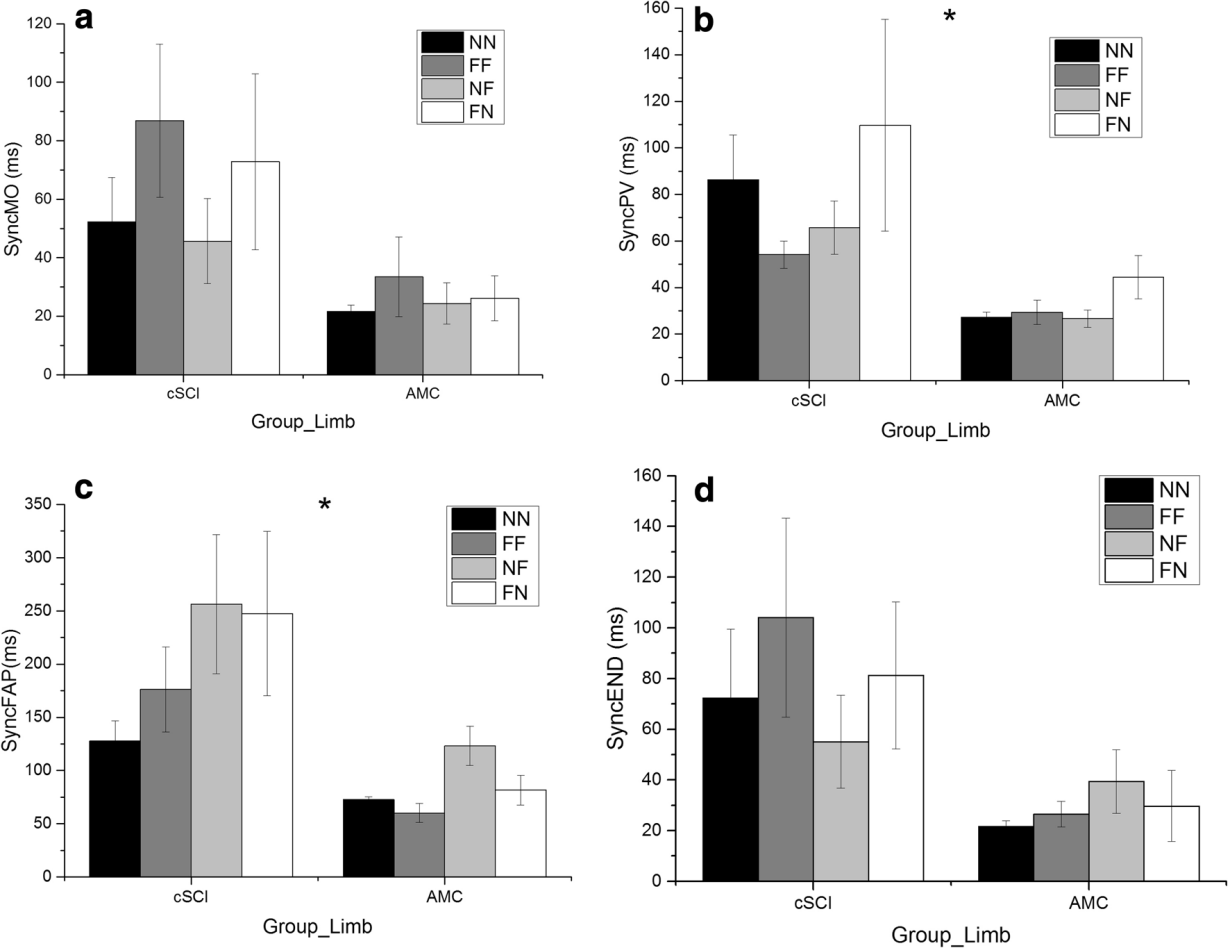
Group means (± standard error) for interlimb synchrony at movement onset (SyncMO) (**a**), at ToPV (SyncPV) (**b**), at FAP_start_ (SyncFAP) (**c**) and at the end of the movement (SyncEND) (**d**) for NN (black), FF (dark grey), NF (light grey) and FN (white) conditions. (*Denotes significant difference between groups)

At movement onset, there was no significant difference between groups [*F*(1,19) = 3.14, *p* > 0.05, *η*^2^ = 0.14] despite variance seen in participants with a cSCI (Fig. 4a). At ToPV [*F*(1,19) = 8.66, *p* < 0.01, *η*^2^ = 0.31] (Fig. 4b) and FAP_start_ [*F*(1,18) = 7.50, *p* < 0.05, *η*^2^ = 0.30] (Fig. 4c), participants with a cSCI (PV 76 ms, FAP 198 ms) were less synchronous than AMC (PV 32 ms, FAP 82 ms). However, at the end of the movement, the main effect of group did not reach significance [*F*(1,20) = 2.14, *p* > 0.05, *η*^2^ = 0.10] (Fig. 4d). The main effect of condition did not reach significance at any phase of the movement and no significant interactions emerged.

### EMG

Peak muscle activity in relation to kinematic events occurred closest to the start of the final adjustment phase (FAP_start_) for all muscles tested (see Fig. 2), therefore, this was the focus of the subsequent statistical analyses (Table 2). We also calculated the timing of peak muscle activity in relation to all other kinematic events but for reasons of brevity include these data as Supplementary Table 2.

**Table 2:** Group means for the timing of peak muscle activity (as a percentage of movement time) minus the timing of the start of the final adjustment phase (FAP_start_) (as a percentage of movement time) for each bimanual condition collapsed across limb [difference in timing as a percentage of movement time is presented (%)]

	Group	Anterior deltoid	Biceps brachii	Extensor digitorum superficialis	Triceps brachii
Condition 1—Near Near	cSCI	− 8.79	− 6.97	− 7.54	− 6.38
	AMC	− 9.88	− 9.89	− 4.05	− 8.10
Condition 2—Far Far	cSCI	0.38	− 7.23	− 5.37	− 4.48
	AMC	− 6.00	− 7.72	− 3.32	− 7.66
Condition 3—Near Far	cSCI	5.21	− 8.62	1.66	4.94
	AMC	1.38	− 0.6	3.82	− 2.51
Condition 4—Far Near	cSCI	9.48	0.68	4.74	4.74
	AMC	− 6.02	− 8.09	− 4.73	− 12.27

A positive number indicates that the timing of peak muscle activity occurred after the timing of FAP_start_

There was no significant main effect of group for any of the muscles tested except the triceps brachii [*F*(1,15) = 10.75, *p* < 0.05, *η*^2^ = 0.42] as peak triceps brachii activity was significantly closer to FAP_start_ for participants with a cSCI (*m* = 0.19%) compared to AMC (*m* = − 14.40%).

For the anterior deltoid, there was a significant main effect of condition [*F*(3,45) = 3.29, *p* < 0.05, *η*^2^ = 0.18] as peak anterior deltoid activity was closer to FAP_start_ in condition four (FN = 2.46%) compared to condition one (NN = − 10.52%).

A condition by group interaction emerged for the anterior deltoid [*F*(3,45) = 3,28, *p* < 0.05, *η*^2^ = 0.18], extensor digitorum superficialis [*F*(3,45) = 3.12, *p* < 0.05, *η*^2^ = 0.17] and triceps brachii [*F*(3,45) = 3.13, *p* < 0.05, *η*^2^ = 0.17]. Repeated-measures ANOVAs for each group and each muscle revealed no significant main effect of condition. Independent *T* tests revealed that for the symmetrical trials (NN and FF) there was no significant difference between groups for any of the muscles. However, for the asymmetrical trials (NF and FN), there was a significant difference between groups. In condition three (NF) peak anterior deltoid, extensor digitorum superficialis and triceps brachii activity were after FAP_start_ in participants with a cSCI (AD = 5.21%, *E* = 1.65%, *T* = 4.93%) but before FAP_start_ for AMC (AD = − 21.60%, *E* = − 24.22%, *T* = − 32.88%). In condition four (FN) peak triceps brachii and anterior deltoid activity occurred before FAP_start_ for participants with a cSCI (*T* = 4.74%, AD = 9.47%) but after FAP_start_ in AMC (*T* = − 12.27%, AD = − 6.02%).

In terms of agonist–antagonist muscle activity patterns, there was a significant main effect of group [*F*(1,12) = 14.16, *p* < 0.05, *η*^2^ = 0.54] as the time of peak triceps brachii activity and lowest biceps brachii activity was less coupled in participants with a cSCI (*m* = 24.59%) than AMC (*m* = 3.14%).

## Discussion

This study explored the effect of task symmetry (object distance) on bimanual reach-to-grasp movements in individuals with cSCI and how this differs to AMC. Individuals with a cSCI took longer (duration) to complete their movements, reached lower peak velocities, spent a longer proportion of the movement in the deceleration and final adjustment phases (as a percentage of movement time) and made more adjustments to their reach-to-grasp movements (Figs. 1, 2 and 3a–f). Interlimb synchrony at movement onset and end of the movement did not differ between groups, but the participants with a cSCI were less synchronous than the AMC during the reach (at ToPV and FAP_start_), although no more affected by task symmetry (Fig. 4a–d). The timing of peak muscle activity was similar for both groups (closest to FAP_start_) and there were no significant differences between the limbs (Table 2).

The increased reliance on the deceleration phase (in line with hypothesis 1) to successfully reach and grasp the object agrees with previous unimanual research comparing participants with a cSCI and non-injured control participants (Laffont et al. 2000; Hoffmann et al. 2006; de los Reyes-Guzmán et al. 2010; Mateo et al. 2013; Britten et al. 2017). One plausible explanation is that due to declines in proprioceptive abilities (Gordon et al. 1995) participants with a cSCI produce a prolonged deceleration phase and prolonged final adjustment phase to correct errors when they can visually fixate the limb and object in relation to one another, i.e. late in the movement (Coats and Wann 2012). This is further supported by participants with a cSCI making more adjustments in both the approach and the final adjustment phase. The increased number of adjustments is also consistent with previous unimanual research as participants with a cSCI showed an increase in the number of small but multiple accelerations, i.e. adjustments, of the upper limb compared to non-injured control participants (Koshland et al. 2005; Britten et al. 2017).

In relation to hypothesis two, at movement onset, there was no significant difference in interlimb synchrony between cSCI and AMC (Fig. 4). However, at ToPV and FAP_start_, interlimb synchrony was reduced for participants with a cSCI when compared to AMC. This may be due to loss of sensory function after cSCI, particularly proprioception (Gordon et al. 1995). A loss of sensory control can subsequently increase reliance on visual feedback as suggested in older adults (Sosnoff and Newell 2006; Coats and Wann 2012), which naturally produces asynchrony between the limbs as it is not possible to fixate both limbs/objects at the same time (Riek et al. 2003; Mason and Bruyn 2009). This assumption would need further testing to specifically investigate the importance of visual feedback following cSCI when performing reach-to-grasp movements. However, an increased reliance on visual feedback during gait has already been shown in individuals with a SCI when crossing obstacles (Malik et al. 2017). Interestingly, the difference between groups was not significant by the end of the movement and interlimb asynchrony was reduced [when compared to FAP_start_ (Fig. 4c, d)], which suggests that both groups used the final adjustment phase to reduce the asynchrony between the two limbs despite no specific instruction to do so.

Although peak muscle activity patterns were similar for both groups in all conditions (occurring closest to FAP_start_ compared to other kinematic events), peaks were after FAP_start_ for cSCI (positive number) and just before FAP_start_ (negative number) for AMC. This suggests that participants with a cSCI may use their peak muscle activity to apply a ‘braking force’ to the upper limb to then correct for errors prior to object pick up, hence the longer final adjustment phase with a greater number of adjustments. The kinematic movement slowing (longer movement time) that occurred in individuals with a cSCI may have arisen due to the reduction in triceps brachii and biceps brachii agonist–antagonist muscle activity following cSCI, as the triceps brachii serves to extend the elbow whilst the biceps brachii acts to stop further extension in non-injured participants (Koshland et al. 2005; Hughes et al. 2009). Therefore, the movement slowing may be a strategy adopted by participants with a cSCI, to decrease the reliance on the biceps brachii to stop further extension of the elbow, as faster movements require greater muscle activity to stop the movement (Koshland et al. 2005; Hughes et al. 2009).

With regards to the grasp phase of the movement, there was no significant difference between groups in terms of maximum grasp aperture or when this occurred in the movement. The lack of difference between groups may have occurred due to object size remaining constant, which differs to previous research (Calabro and Perez 2016). However, participants with a cSCI did produce reach-to-grasp movements with less coupled transport and grasp phases than AMC, which agrees with previous unimanual research (Mateo et al. 2013), although none of the participants in the current study had tendinosis.

There were no conditions by group interactions for any of the kinematic parameters, which suggest that participants with a cSCI were no differently affected by task symmetry than AMC. The lack of significant main effect of limb or group by limb interactions suggests that the more impaired limb did not influence the less impaired limb, even in asymmetrical tasks. These findings are surprising considering bilateral deficits following cSCI (Spooren et al. 2009). However, all participants in the current study scored well on the Chedoke Arm and Hand Inventory (Barreca et al. 2004) (see Table 1), and therefore the level of functional bimanual deficit may have been minimal. It could also be that the spared fibres of the CST, spinal interneurons and propriospinal neurons allowed for bimanual control to be maintained (Sasaki et al. 2004; Lemon 2008; Rosenzweig et al. 2009, 2010; Krajacic et al. 2010; Takei and Seki 2013) as participants in this study had sustained an incomplete cSCI.

## Potential limitations and future work

One potential limitation for the present study is the variance in skeletal level between participants with a cSCI. We did explore the data with skeletal level as a covariate for all kinematic and EMG parameters, however, no significant main effects or interactions emerged. Therefore, we do not present that data here as this is likely due to the small number of participants for each skeletal level or some participants sustaining injury over multiple skeletal levels, both of which are a result of the heterogeneity of the SCI population (see Table 1). The inclusion of MRI data would allow for consideration of bilateral neurodegenerative changes following cSCI, e.g. level of injury to the CST or dorsal column of the spinal cord. This would allow for us to explore the effects of differing locations/severity of cSCI to bimanual control.

The longer proportion of the movement spent in the final adjustment phase following cSCI, may have occurred due to detriments in grip force modulation, via disruption of the CST. This is because the direct cortico-motoneuronal network of the CST, as well as premotor spinal interneurons in the cervical spinal cord, have shown activation during the dynamic phase of the precision grip (squeezing the index finger and thumb together), which was required in the current study (Bennett and Lemon 1996; Takei and Seki 2013). To test this in future research, the addition of force transducers on the object surface would give further insight into this control. Research has suggested that maximal grip strength is reduced following cSCI but the effects of this have not been measured in functional tasks (such as picking up objects) (Gomes-Osman et al. 2017). Functional training with peripheral nerve somatosensory stimulation (Gomes-Osman et al. 2017) and epidural stimulation of the cervical spinal cord alone (Lu et al. 2016) have been shown to increase precision grip strength in individuals with chronic tetraplegia. If these interventions were implemented at the acute stage of injury when the greatest neuroplasticity occurs (Curt et al. 2008) the gains in precision grip strength and volitional hand control may be greater and subsequently allow for greater functional independence.

## Conclusion

Overall, these data suggest that although the hands might move less synchronously in the middle stages of reach-to-grasp movements (ToPV to FAP_start_) compared to AMC, a level of bimanual control is retained after cSCI such that participants aim to end the movement in a synchronous fashion without specific instruction to do so (i.e. reducing the asynchrony between the limbs between FAP_start_ and end of the movement to pick both objects up together). Task symmetry does not influence this pattern differently in people with cSCI compared to their non-injured AMC. This study also supports the use of more complex performance measures (Kinematics and surface EMG) to analyse bimanual movements compared to more descriptive clinical/functional scales.

## Electronic supplementary material

Below is the link to the electronic supplementary material.

Supplementary material 1 (DOCX 22 KB)

Supplementary material 2 (DOCX 22 KB)
