# Supplementary material for: The effect of task symmetry on bimanual reach-to-grasp movements after cervical spinal cord injury
Source: Exp Brain Res. 2018 Aug 21;236(11):3101–11. doi: 10.1007/s00221-018-5354-8 (PMC6223837; doi:10.1007/s00221-018-5354-8)
Supplement: Supplementary file 1 — Supplementary material 1 (DOCX 22 KB) [file 221_2018_5354_MOESM1_ESM.docx]

Supplementary table 1: Group and limb means (±standard error) for Movement time (MT), Peak Velocity (PV), Deceleration time (DT; as a percentage of MT), Final Adjustment Phase (FAP; as a percentage of MT), Number of adjustments in the approach phase (NOAA), Number of adjustments in FAP (NOAF), Maximum grasp aperture (MGA), the time at which MGA occurred during MT (tMGA) and the coupling of the grasp and transport phase (TrG) for each bimanual condition.

| **Condition one – Near Near** | | | | | | | | | |
| --- | --- | --- | --- | --- | --- | --- | --- | --- | --- |
| Preferred/Less impaired limb | | | | | | | | | |
|  | MT (ms) | PV (mm/s) | DT (%) | FAP(%) | NOAA | NOAF | MGA (cm) | tMGA (%) | TrG (ms) |
| cSCI | 1722.73  (235.66) | 479.44  (44.08) | 75.30  (1.93) | 30.34  (4.36) | 3.68  (0.64) | 9.13  (3.80) | 9.95  (0.32) | 49.48  (3.02) | 220.18  (55.17) |
| AMC | 960.34  (52.96) | 613.77  (40.83) | 68.40  (1.52) | 21.29  (3.14) | 1.67  (0.50) | 2.76  (0.75) | 9.39  (0.13) | 57.43  (2.20) | 108.48  (17.64) |
| Non-preferred/More impaired limb | | | | | | | | | |
|  | MT (ms) | PV (mm/s) | DT (%) | FAP(%) | NOAA | NOAF | MGA (cm) | tMGA (%) | TrG (ms) |
| cSCI | 1776.84  (271.87) | 489.92  (47.32) | 76.27  (1.98) | 32.68  (6.55) | 4.68  (0.91) | 9.79  (4.49) | 9.54  (0.35) | 53.72  (2.27) | 465.42  (189.71) |
| AMC | 977.88  (56.21) | 608.56  (47.55) | 67.88  (1.73) | 19.30  (1.73) | 1.60  (1.72) | 2.28  (1.73) | 9.70  (1.72) | 55.60  (1.73) | 98.01  (19.58) |
| **Condition two – Far Far** | | | | | | | | | |
| Preferred/Less impaired limb | | | | | | | | | |
|  | MT (ms) | PV (mm/s) | DT (%) | FAP(%) | NOAA | NOAF | MGA (cm) | tMGA (%) | TrG (ms) |
| cSCI | 1966.05  (272.07) | 572.44  (58.70) | 75.49  (1.90) | 32.94  (4.33) | 4.52  (0.95) | 8.22  (2.15) | 9.79  (0.23) | 55.07  (4.92) | 384.83  (52.80) |
| AMC | 1023.68  (50.59) | 798.03  (43.04) | 68.95  (1.17) | 17.82  (2.92) | 1.33  (0.30) | 2.29  (0.50) | 9.62  (0.17) | 57.98  (2.01) | 130.88  (12.17) |
| Non-preferred/More impaired limb | | | | | | | | | |
|  | MT (ms) | PV (mm/s) | DT (%) | FAP(%) | NOAA | NOAF | MGA (cm) | tMGA (%) | TrG (ms) |
| cSCI | 1987.53  (293.37) | 559.11  (62.50) | 76.25  (1.53) | 31.85  (4.63) | 4.95  (1.14) | 10.19  (3.21) | 9.47  (0.37) | 61.79  (5.32) | 520.52  (83.75) |
| AMC | 1054.41  (55.37) | 768.14  (51.58) | 68.63  (1.18) | 16.43  (2.20) | 1.57  (0.42) | 1.98  (0.33) | 10.08  (0.23) | 56.50  (2.15) | 125.02  (17.56) |
| **Condition three – Near Far** | | | | | | | | | |
| Preferred/Less impaired limb | | | | | | | | | |
|  | MT (ms) | PV (mm/s) | DT (%) | FAP(%) | NOAA | NOAF | MGA (cm) | tMGA (%) | TrG (ms) |
| cSCI | 1658.60  (186.97) | 430.84  (56.86) | 77.83  (1.97) | 35.81  (3.98) | 3.66  (0.65) | 7.44  (1.35) | 8.89  (0.82) | 53.66  (2.15) | 251.23  (67.71) |
| AMC | 1018.3  (47.80) | 595.00  (69.81) | 71.51  (1.33) | 26.24  (2.10) | 1.34  (0.40) | 3.21  (0.36) | 8.81  (0.84) | 54.56  (1.29) | 94.48  (19.57) |
| Non-preferred/More impaired limb | | | | | | | | | |
|  | MT (ms) | PV (mm/s) | DT (%) | FAP(%) | NOAA | NOAF | MGA (cm) | tMGA (%) | TrG (ms) |
| cSCI | 1694.16  (198.11) | 544.76  (73.78) | 75.28  (1.66) | 29.25  (6.60) | 4.86  (1.24) | 6.23  (1.44) | 8.58  (0.84) | 59.31  (2.51) | 330.54  (67.71) |
| AMC | 1033.14  (53.07) | 713.93  (82.64) | 69.63  (1.45) | 14.93  (2.07) | 1.39  (0.23) | 1.69  (0.32) | 9.08  (0.86) | 59.31  (1.71) | 140.41  (21.52) |
| **Condition four – Far Near** | | | | | | | | | |
| Preferred/Less impaired limb | | | | | | | | | |
|  | MT (ms) | PV (mm/s) | DT (%) | FAP(%) | NOAA | NOAF | MGA (cm) | tMGA (%) | TrG (ms) |
| cSCI | 1751.70  (186.37) | 570.73  (56.79) | 74.30  (1.81) | 34.65  (3.94) | 2.68  (0.52) | 7.28  (1.45) | 9.92  (0.27) | 48.67  (2.62) | 193.63  (52.22) |
| AMC | 977.67  (58.60) | 714.63  (75.95) | 67.87  (1.65) | 13.61  (1.65) | 1.28  (0.26) | 1.41  (1.65) | 8.74  (0.81) | 60.3  (1.65) | 131.31  (23.76) |
| Non-preferred/More impaired limb | | | | | | | | | |
|  | MT (ms) | PV (mm/s) | DT (%) | FAP(%) | NOAA | NOAF | MGA (cm) | tMGA (%) | TrG (ms) |
| cSCI | 1778.04  (192.66) | 464.17  (47.01) | 75.71  (1.85) | 38.30  (3.90) | 4.28  (0.93) | 9.20  (2.27) | 9.11  (0.52) | 51.01  (3.74) | 312.08  (73.35) |
| AMC | 984.36  (58.85) | 569.01  (70.76) | 70.12  (1.64) | 21.50  (2.83) | 1.36  (0.16) | 2.53  (0.53) | 9.11  (0.85) | 55.4  (2.33) | 84.35  (24.02) |
