# Supplementary material for: The effect of task symmetry on bimanual reach-to-grasp movements after cervical spinal cord injury
Source: Exp Brain Res. 2018 Aug 21;236(11):3101–11. doi: 10.1007/s00221-018-5354-8 (PMC6223837; doi:10.1007/s00221-018-5354-8)
Supplement: Supplementary file 2 — Supplementary material 2 (DOCX 22 KB) [file 221_2018_5354_MOESM2_ESM.docx]

Supplementary table 2: Group and limb means for the difference in timing of peak muscle activity (as a percentage of movement time) and the timing of kinematic events (as a percentage of movement time) for each bimanual condition (difference in timing as a percentage of movement time is presented). A positive value indicates that peak muscle activity occurred after the kinematic event. The nearest kinematic event is in **bold.**

| **Condition one – Near Near** | | | | | | | | | | | | | | | | |
| --- | --- | --- | --- | --- | --- | --- | --- | --- | --- | --- | --- | --- | --- | --- | --- | --- |
| Preferred/Less impaired limb | | | | | | | | | | | | | | | | |
|  | Anterior Deltoid | | | | Biceps Brachii | | | | Extensor Digitorum Superficialis | | | | Triceps Brachii | | | |
|  | MO | ToPV | FAP_start_ | END | MO | ToPV | FAP_start_ | END | MO | ToPV | FAP_start_ | END | MO | ToPV | FAP_start_ | END |
| cSCI | 63.17 | 20.11 | **-7.63** | -36.83 | 60.96 | 17.91 | **-9.84** | -39.04 | 53.70 | 14.96 | **-10.02** | -36.30 | 58.71 | 19.97 | **-5.00** | -31.29 |
| AMC | 79.22 | 25.74 | **-5.28** | -20.78 | 74.77 | 21.29 | **-9.74** | -25.23 | 82.07 | 28.59 | **-2.43** | -17.93 | 76.14 | 22.65 | **-8.37** | -23.86 |
| Non-preferred/More impaired limb | | | | | | | | | | | | | | | | |
|  | Anterior Deltoid | | | | Biceps Brachii | | | | Extensor Digitorum Superficialis | | | | Triceps Brachii | | | |
|  | MO | ToPV | FAP_start_ | END | MO | ToPV | FAP_start_ | END | MO | ToPV | FAP_start_ | END | MO | ToPV | FAP_start_ | END |
| cSCI | 71.19 | 19.25 | **-9.94** | -28.81 | 77.02 | 25.09 | **-4.10** | -22.98 | 76.08 | 24.14 | **-5.05** | -23.92 | 73.37 | 21.43 | **-7.76** | -26.63 |
| AMC | 72.25 | 18.20 | **-14.48** | -27.75 | 76.69 | 22.64 | **-10.04** | -23.31 | 81.07 | 27.01 | **-5.66** | -18.93 | 78.91 | 24.86 | **-7.82** | -21.09 |
| **Condition two – Far Far** | | | | | | | | | | | | | | | | |
| Preferred/Less impaired limb | | | | | | | | | | | | | | | | |
|  | Anterior Deltoid | | | | Biceps Brachii | | | | Extensor Digitorum Superficialis | | | | Triceps Brachii | | | |
|  | MO | ToPV | FAP_start_ | END | MO | ToPV | FAP_start_ | END | MO | ToPV | FAP_start_ | END | MO | ToPV | FAP_start_ | END |
| cSCI | 78.67 | 37.20 | **4.81** | -21.33 | 70.24 | 28.76 | **-3.63** | -29.76 | 75.60 | 34.12 | **1.73** | -24.41 | 73.67 | 32.19 | **-0.20** | -26.33 |
| AMC | 76.67 | 30.96 | **-5.51** | -23.33 | 67.01 | 21.30 | **-4.14** | -32.99 | 71.01 | 25.29 | **-0.15** | -28.99 | 70.09 | 24.37 | **-1.07** | -29.91 |
| Non-preferred/More impaired limb | | | | | | | | | | | | | | | | |
|  | Anterior Deltoid | | | | Biceps Brachii | | | | Extensor Digitorum Superficialis | | | | Triceps Brachii | | | |
|  | MO | ToPV | FAP_start_ | END | MO | ToPV | FAP_start_ | END | MO | ToPV | FAP_start_ | END | MO | ToPV | FAP_start_ | END |
| cSCI | 83.43 | 28.45 | **-4.06** | -16.57 | 76.67 | 21.68 | **-10.82** | -23.33 | 75.02 | 20.03 | **-12.47** | -24.98 | 78.75 | 23.75 | **-8.75** | -21.26 |
| AMC | 83.63 | 28.83 | **-6.49** | -16.37 | 78.82 | 24.02 | **-11.29** | -21.18 | 83.63 | 28.82 | **-6.48** | -99.45 | 75.86 | 21.06 | **-14.25** | -24.14 |
| **Condition three – Near Far** | | | | | | | | | | | | | | | | |
| Preferred/Less impaired limb | | | | | | | | | | | | | | | | |
|  | Anterior Deltoid | | | | Biceps Brachii | | | | Extensor Digitorum Superficialis | | | | Triceps Brachii | | | |
|  | MO | ToPV | FAP_start_ | END | MO | ToPV | FAP_start_ | END | MO | ToPV | FAP_start_ | END | MO | ToPV | FAP_start_ | END |
| cSCI | 80.40 | 38.88 | **7.04** | -19.60 | 66.12 | 24.59 | **-7.24** | -33.88 | 73.32 | 31.79 | **-0.04** | -26.68 | 78.37 | 36.85 | **5.02** | -21.63 |
| AMC | 94.03 | 23.99 | **-5.06** | -14.03 | 97.21 | 20.81 | **-8.25** | -17.21 | 93.93 | 24.09 | **4.97** | -13.93 | 94.81 | 33.21 | **-4.16** | -4.81 |
| Non-preferred/More impaired limb | | | | | | | | | | | | | | | | |
|  | Anterior Deltoid | | | | Biceps Brachii | | | | Extensor Digitorum Superficialis | | | | Triceps Brachii | | | |
|  | MO | ToPV | FAP_start_ | END | MO | ToPV | FAP_start_ | END | MO | ToPV | FAP_start_ | END | MO | ToPV | FAP_start_ | END |
| cSCI | 82.70 | 39.32 | **3.38** | -17.30 | 69.34 | 25.96 | **-9.99** | -30.66 | 82.67 | 39.29 | **3.35** | -17.33 | 84.17 | 40.79 | **4.85** | -15.83 |
| AMC | 90.84 | 19.20 | **7.82** | -10.84 | 90.06 | 19.98 | **7.05** | -10.06 | 95.68 | 24.36 | **2.66** | -5.68 | 97.49 | 35.55 | **-2.51** | -5.53 |
| **Condition four – Far Near** | | | | | | | | | | | | | | | | |
| Preferred/Less impaired limb | | | | | | | | | | | | | | | | |
|  | Anterior Deltoid | | | | Biceps Brachii | | | | Extensor Digitorum Superficialis | | | | Triceps Brachii | | | |
|  | MO | ToPV | FAP_start_ | END | MO | ToPV | FAP_start_ | END | MO | ToPV | FAP_start_ | END | MO | ToPV | FAP_start_ | END |
| cSCI | 81.85 | 36.84 | **12.50** | -18.15 | 67.97 | 22.96 | **-1.38** | -32.03 | 75.18 | 30.17 | **5.83** | -24.82 | 73.53 | 28.51 | **4.17** | -26.47 |
| AMC | 86.78 | 32.13 | **-3.63** | -13.22 | 80.16 | 25.51 | **-10.25** | -19.84 | 83.52 | 28.87 | **-6.89** | -16.48 | 76.17 | 21.52 | **-14.23** | -23.83 |
| Non-preferred/More impaired limb | | | | | | | | | | | | | | | | |
|  | Anterior Deltoid | | | | Biceps Brachii | | | | Extensor Digitorum Superficialis | | | | Triceps Brachii | | | |
|  | MO | ToPV | FAP_start_ | END | MO | ToPV | FAP_start_ | END | MO | ToPV | FAP_start_ | END | MO | ToPV | FAP_start_ | END |
| cSCI | 75.95 | 35.26 | **6.46** | -24.05 | 72.23 | 31.54 | **2.74** | -27.77 | 73.19 | 32.45 | **3.65** | -26.86 | 74.80 | 34.11 | **5.31** | -25.20 |
| AMC | 77.30 | 23.66 | **-8.41** | -22.70 | 79.80 | 26.16 | **-5.92** | -20.20 | 83.16 | 29.52 | **-2.56** | -16.84 | 75.42 | 21.78 | **-10.30** | -24.58 |

MO= movement onset, ToPV= time of peak velocity, FAP_start_= start of the final adjustment phase and END= end of the movement.
